# Mini-laparotomic-assisted laparoscopic radical hysterectomy: an innovative technique for cervical cancer surgery—a case series

**DOI:** 10.1007/s00404-025-08224-y

**Published:** 2025-10-22

**Authors:** Ala Aiob, Dina Gumin, Avishalom Sharon, Lior Lowenstein

**Affiliations:** 1 Department of Obstetrics and Gynecology, Galilee Medical Center, Nahariya, Israel; 2 Azrieli Faculty of Medicine, Bar Ilan University, Safed, Israel

**Keywords:** Cervical cancer, Radical hysterectomy, Minimally invasive surgery, Protective surgical techniques, Tumor spillage prevention

## Abstract

**Purpose:**

To assess the feasibility, safety, and short-term surgical outcomes of mini-laparotomic-assisted laparoscopic radical hysterectomy in the treatment of early-stage cervical cancer. This pilot feasibility case series is the first to provide a detailed description of the technique and its initial clinical results.

**Methods:**

A retrospective case series of seven women with early-stage cervical cancer underwent mini-laparotomic-assisted laparoscopic radical hysterectomy at a single tertiary care center between November 2023 and October 2024. The surgical procedure included laparoscopic pelvic lymphadenectomy, radical hysterectomy, and salpingectomy, followed by colpotomy and uterine extraction through a mini-laparotomy (4–8 cm). Data on baseline characteristics, intraoperative parameters, and postoperative outcomes were collected and analyzed retrospectively.

**Results:**

The median age was 50 years (range 42–76), and the median BMI was 27.9 kg/m^2^ (range 20–43). Histological subtypes included five cases of squamous cell carcinoma, one case of adenocarcinoma, and one case of adenosquamous carcinoma. Pathological staging revealed IB2 in three patients, IA1 in two, and one each for IB1 and IA2. The median operative time was 345 min (range 295–395), and the median estimated blood loss was 500 mL (range 200–700). No intraoperative or postoperative complications were reported.

**Conclusion:**

Mini-laparotomic-assisted laparoscopic radical hysterectomy appears to be a feasible and safe surgical option for early-stage cervical cancer. This technique combines the oncologic rigor of open surgery with the advantages of minimally invasive methods, addressing significant limitations of conventional laparoscopy, including tumor manipulation and intracorporeal colpotomy performed under CO₂ pneumoperitoneum. As the initial report describing this approach, the findings support its potential as an effective alternative to the traditional open radical hysterectomy. Further studies involving larger cohorts and long-term follow-up are needed to validate its oncologic and perioperative benefits.

## What does this study adds to the clinical work

**Table:** 

Mini-laparotomic-assisted laparoscopic radical hysterectomy is a feasible, safe, and potentially oncologically sound technique for managing cervical cancer. By combining the benefits of minimally invasive surgery with strategic modifications that address the oncologic concerns of conventional laparoscopy, such as avoiding uterine manipulators and performing endoscopic intracorporeal colpotomy with CO₂ insufflation, this hybrid approach provides a promising alternative to open radical hysterectomy. Further validation in larger, prospective studies is necessary.	

## Introduction

Mini-laparotomic-assisted laparoscopic radical hysterectomy is a hybrid surgical approach in which most of the radical hysterectomy and pelvic lymphadenectomy are performed laparoscopically. However, specimen removal and some critical steps (such as colpotomy or parametrial dissection) are completed through a small laparotomy incision, typically 4–8 cm. This technique aims to combine the perioperative benefits of minimally invasive surgery (MIS)—such as reduced blood loss, shorter hospital stay, and faster recovery—with the oncologic safety of open surgery by minimizing the risk of tumor spillage during specimen extraction and colpotomy. Current national and international guidelines recommend surgical treatment for early-stage cervical cancer, but the extent of surgery depends on the specific stage and risk factors. Radical hysterectomy is indicated for stages IB1–IIA1, whereas less radical surgery may be sufficient for IA1 disease without lymphovascular space invasion [1]. While ARH has been the standard approach for many years, it is associated with higher rates of complications, including bladder dysfunction, extended hospital stays, and increased postoperative infections [2–4]. In contrast, laparoscopic radical hysterectomy (LRH) offers advantages such as reduced intraoperative blood loss and shorter recovery times, which have led to its widespread adoption as a standard approach for early-stage cervical cancer [5–7].

However, the 2018 publication of the Laparoscopic Approach to Cervical Cancer (LACC) trial significantly influenced surgical protocols by revealing notably lower overall survival (OS) and disease-free survival (DFS) rates in patients undergoing LRH compared to ARH, raising substantial concerns [8]. Subsequent studies corroborated these findings, suggesting a concerning trend of higher recurrence and mortality rates associated with minimally invasive procedures [9].

These discrepancies in outcomes highlight potential technical factors that influence oncologic results, particularly the use of uterine manipulators and techniques such as intracorporeal colpotomy, which may increase the risk of tumor disruption [10, 11]. To address these challenges, innovative techniques have been proposed that combine the benefits of laparoscopy with the oncologic safety of open surgery [11].

This pilot feasibility case series presents a novel surgical approach: mini-laparotomic-assisted laparoscopic radical hysterectomy. This technique aims to optimize surgical efficacy while reducing the oncologic risks often seen with minimally invasive methods. To the best of our knowledge, this is the first study to describe the surgical procedure in detail and to report the short-term surgical outcomes associated with its implementation.

## Material and methods

This study received institutional review board approval (NHR- 0216–24). It involved a retrospective analysis of seven women who underwent mini-laparotomic-assisted laparoscopic radical hysterectomy for the staging and treatment of early-stage cervical cancer at the Gynecologic Oncology Services of Galilee Medical Center, Israel, between November 2023 and October 2024.

Two primary surgeons, Dr. A.A. and Dr. L.L., performed all surgeries with support from residents during the procedures. Comprehensive data were retrieved retrospectively from patients' electronic health records, including sociodemographic information, a history of previous abdominal surgeries, any concomitant surgical procedures, and oncologic details such as preoperative disease stage, histological type, and final FIGO stage.

Intraoperative data collected included anesthesia time, total operating time (defined as the duration from the initial incision to skin closure), estimated blood loss, and any intraoperative complications encountered. Postoperative follow-up assessments focused on urinary and gastrointestinal symptoms, as well as the length of hospitalization.

This systematic approach ensures a comprehensive evaluation of both operative outcomes and patient recovery following the mini-laparotomic-assisted laparoscopic radical hysterectomy, contributing to the understanding of its feasibility and safety in managing early-stage cervical cancer.

Surgical technique: The patient was positioned under general anesthesia in a low lithotomy position with a slight Trendelenburg tilt. Pneumatic compression stockings were applied for thromboprophylaxis, and a urinary catheter was placed for bladder management. Prophylactic antibiotics were administered preoperatively. Four trocars were placed to provide optimal access and visualization: a 10–12 mm trocar at the umbilicus for the laparoscope and an additional 5 mm trocar in each lower quadrant and the midline.

The procedure started with dividing the round ligaments, followed by dissecting the broad ligament to reveal the pelvic blood vessels and the paravesical and pararectal spaces. Sentinel lymph node mapping was performed with indocyanine green (ICG) to identify sentinel nodes, which were then sent for frozen section analysis. Upon confirmation of no malignant cells in the sentinel nodes, we conducted a systematic pelvic lymphadenectomy, which included the obturator, internal, external, and common iliac nodes. Next, the uterine arteries were identified, isolated, and ligated at their origin from the internal iliac arteries or at the level of the ureter. The ureters were dissected and mobilized away from the cervix and vaginal cuff and tracked to their insertion into the bladder [12]. The parametrium and paracolpos were dissected to the level of the ureter or the origin of the uterine artery to ensure thorough removal of potential cancer spread while preserving autonomic nerves essential for bladder and bowel function.

Upon reaching the point of colpotomy, the laparoscopic procedure was halted. A mini-laparotomy (4–8 cm, low transverse incision) was performed to facilitate a standard colpotomy and ensure the safe extraction of the uterus. The vaginal cuff was then securely closed, and the abdominal wall was sutured as usual. A cystoscopy was conducted in all patients to confirm that there was no ureteral or bladder injury.

## Results

Between November 2023 and October 2024, seven women underwent mini-laparotomic-assisted laparoscopic radical hysterectomy for the staging and treatment of cervical cancer. The baseline characteristics of the patients are presented in Table 1. The median age was 50 years (42–76), and the median BMI was 27.9 kg/m^2^ (20–43). Histopathological diagnoses included squamous cell carcinoma (SCC) in five patients, adenocarcinoma in one, and adenosquamous carcinoma in another.

**Table 1:** Sociodemographic data and medical history

Age (years), median (range)	50 (42–76)
Parity, median (range)	3 (1–5)
BMI (kg/m2), median (range)	27(20–43)
Hypertension, number (%)	3 (43%)
Hypothyroidism, number (%)	1 (15%)
Cardiac disease, number (%)	2 (29%)
Diabetes, number (%)	2 (29%)
Previous pelvic/abdominal surgery, number (%)	3 (43%)

Six women underwent preoperative conization, whereas one patient with a visible cervical mass had a punch biopsy demonstrating 4 mm stromal invasion. Preoperative PET-CT and MRI confirmed early-stage cervical cancer in all cases. After intraoperative confirmation of negative lymph nodes through frozen section analysis, all patients underwent radical hysterectomy.

According to our institutional pathology reports, which follow NCCN guidelines, parametrial involvement is reported in binary terms (present or absent) rather than by measuring the parametrial dimensions. In all cases, the parametrium was free of tumor, and the neoplasm was not close to the resection margin. The final pathology revealed stage IB2 disease in three patients, stage IA1 in two, stage IB1 in one, and stage IA2 in one (Table 2).

**Table 2:** Indications for surgery and final staging according to FIGO 2018

Patient	Indication	Preoperative Stage	Final Stage
1	SCC	1B1*	1A2
2	ACC	1B2	1B2
3	ASCC	1B1*	1A1
4	SCC	1B2	1B2
5	SCC	1B1	1B1
6	SCC	1A2	1B2
7	SCC	1B1*	1A1

*SCC* Squamous cell carcinoma, *ACC* Adenocarcinoma of the cervix, *ASC* Adenosquamous carcinoma of the cervix

^*^Incompletely excised tumor

In all patients, the surgical procedure was initiated laparoscopically, and each patient underwent a radical hysterectomy, bilateral salpingectomy, pelvic lymph node dissection, and parametrectomy, followed by a transition to mini-laparotomy for colpotomy and uterine extraction. Bilateral oophorectomy was performed in three cases.

Table 3 summarizes the operational and postoperative details. The median operative time was 345 min (295–395), and the median total anesthesia time was 414 min (366–452). The median estimated blood loss was 500 mL (200–700 mL). No intraoperative or major postoperative complications were observed; however, one patient experienced lymphatic leakage, which resolved without intervention.

**Table 3:** Intraoperative and hospital postoperative data of mini-laparotomic-assisted laparoscopic radical hysterectomy

Anesthesia time, minutes, median (range)	414 (366–452)
Total operative time, minutes, median (range)	345 (295 -395)
Blood loss (mL) median (range)	500 (200–700)
Concomitant surgeries:	
Bilateral salpingo-oophorectomy, number (%)	3 (43%)
Cystoscopy, number (%)	7 (100%)
Intraoperative complications, number (%)	0
Conversion, number (%)	0
Length of hospital stay, median (days)	4 (2–6)
Postoperative complications (during hospitalization), number (%)	1 (14%)

## Discussion

Cervical cancer is the most prevalent gynecological malignancy worldwide, especially in developing countries, where it poses a significant threat to women’s health [13]. For early-stage cervical cancer, radical hysterectomy remains the standard surgical treatment, with 5 year disease-free survival (DFS) rates exceeding 90% [14, 15]. However, the long-term oncologic outcomes of various surgical techniques for radical hysterectomy remain a topic of debate [16].

The LACC trial revealed significantly worse outcomes for minimally invasive surgery (MIS), including a nearly fourfold increase in recurrence risk, cancer-specific mortality, and a sixfold increase in all-cause mortality [8]. The LACC 2024 long-term results [17], along with other epidemiological and meta-analysis studies, have reaffirmed these findings [9, 18]. As a result, open radical hysterectomy has become the recommended approach for early-stage cervical cancer, supported by a category-one recommendation from the National Comprehensive Cancer Network (NCCN) [19].

The inferior outcomes associated with MIS have been attributed to several factors. One key issue is the use of uterine manipulators, which may promote tumor dissemination into the vagina or peritoneal cavity [10, 20–22]. Another concern is exposure to carbon dioxide (CO2) insufflation during MIS, which may facilitate tumor spillage and growth [23, 24]. A study by Kong et al. demonstrated significantly higher recurrence rates with intracorporeal colpotomy compared to vaginal colpotomy (16% vs. 5%), with most recurrences manifesting as intraperitoneal carcinomatosis [25].

Emerging evidence supports using protective surgical techniques to optimize MIS outcomes. Avoiding uterine manipulators and employing tumor-isolating colpotomy techniques have been shown to result in DFS and OS rates comparable to open radical hysterectomy [11]. Additional strategies, such as preoperative conization to reduce tumor volume and prevent spillage, have been associated with decreased recurrence risks [26, 27]. Furthermore, techniques like vaginal colpotomy with tumor isolation via a vaginal cuff and abdominal procedures performed without CO2 insufflation have demonstrated promise in minimizing tumor dissemination and improving oncologic outcomes [18, 28].

In response to these findings, we propose a novel hybrid surgical technique: mini-laparotomic-assisted laparoscopic radical hysterectomy. This approach integrates the oncologic safety of open surgery with the perioperative advantages of laparoscopy. It eliminates the need for uterine manipulators and avoids intracorporeal colpotomy under CO2 pneumoperitoneum. A small, low transvers abdominal incision is used for traditional colpotomy and to facilitate uterine extraction, preserving the tactile feedback of open surgery while leveraging the minimally invasive benefits of laparoscopy.

The strength of this study lies in its introduction of a novel surgical technique designed to address the oncologic limitations of MIS while preserving its well-established perioperative benefits. To the best of our knowledge, this is the first report in the literature to detail this hybrid approach. By eliminating the use of uterine manipulators and avoiding intracorporeal colpotomy under CO₂ pneumoperitoneum, this technique reduces several procedural factors that have been implicated in tumor dissemination, thereby enhancing the oncologic safety profile of minimally invasive surgery. Furthermore, this case series demonstrates the feasibility and safety of the approach, with no intraoperative or postoperative complications, underscoring its potential to enhance patient recovery and reduce surgical morbidity.

However, the study’s primary limitation is the small sample size, which restricts generalizability and prevents robust statistical analysis. Additionally, the retrospective design introduces potential bias due to patient selection and data collection. Another limitation is the lack of long-term follow-up data on recurrence and survival, which are essential for evaluating the technique’s oncologic safety, as well as the inclusion of patients with final stage IA1 disease. Although their surgical management was clinically justified due to positive margins on preoperative conization, their inherently favorable prognosis limits the ability to assess the true oncologic safety of this approach. Another limitation of our study is that parametrial size was not measured in millimeters but only reported in a binary form (tumor present or absent), which may restrict the ability to assess the radicality of hysterectomy specimens fully.

It is important to mention that our reported operative time was longer than what is described in the literature for both laparoscopic and abdominal radical hysterectomy. This prolongation was partly attributable to technical challenges in women with high BMI (particularly the patient with a BMI of 43, where colpotomy required additional time), intraoperative frozen section delays, the learning curve associated with introducing a new surgical technique, and the routine performance of cystoscopy after each case. We acknowledge that longer operative times are generally linked to higher rates of postoperative complications. However, despite the increased duration, we experienced few postoperative complications in our series, and we believe that the hybrid approach provides an advantage over abdominal radical hysterectomy, especially in reducing wound-related morbidity, blood loss, and recovery time. Finally, since this study was conducted at a single center, the findings may not necessarily apply to other institutions or surgical teams with varying levels of expertise [29].

In conclusion, this case series is the first report to describe the surgical technique and to highlight the safety and feasibility of mini-laparotomic-assisted laparoscopic radical hysterectomy for early-stage cervical cancer. By addressing key oncologic concerns of MIS, such as tumor manipulation and intracorporeal colpotomy performed with CO₂ pneumoperitoneum, this hybrid approach presents a promising alternative to open radical hysterectomy. While the short-term outcomes are encouraging, prospective and larger studies with long-term follow-up are needed to validate their oncologic safety and efficacy. If confirmed, this approach could establish a new standard for the surgical management of early-stage cervical cancer, balancing oncologic safety with perioperative benefits.

## Data Availability

All the data generated or analyzed during this study are included in this manuscript. Further inquiries can be directed to the corresponding author.

## References

[1] Tewari KS (2025) Cervical cancer. N Engl J Med 392(1):56–71. 10.1056/NEJMra2404457 39752299 10.1056/NEJMra2404457

[2] Roy M, Plante M, Renaud M-C (2005) Laparoscopically assisted vaginal radical hysterectomy. Best Pract Res Clin Obstet Gynaecol 19(3 SPEC):377–386. 10.1016/j.bpobgyn.2004.12.001 15985253 10.1016/j.bpobgyn.2004.12.001

[3] Nam J-H, Park J-Y, Kim D-Y, Kim J-H, Kim Y-M, Kim Y-T (2012) Laparoscopic versus open radical hysterectomy in early-stage cervical cancer: long-term survival outcomes in a matched cohort study. Ann Oncol 23(4):903–911. 10.1093/annonc/mdr360 21841155 10.1093/annonc/mdr360

[4] Malzoni M, Tinelli R, Cosentino F, Fusco A, Malzoni C (2009) Total laparoscopic radical hysterectomy versus abdominal radical hysterectomy with lymphadenectomy in patients with early cervical cancer: our experience. Ann Surg Oncol 16(5):1316–1323. 10.1245/s10434-009-0342-7 19224286 10.1245/s10434-009-0342-7

[5] Wang Y, Deng L, Xu H et al (2015) Laparoscopy versus laparotomy for the management of early stage cervical cancer. BMC Cancer 15:928. 10.1186/s12885-015-1818-4 26596955 10.1186/s12885-015-1818-4 PMC4657298

[6] Cao T, Feng Y, Huang Q et al (2015) Prognostic and safety roles in laparoscopic versus abdominal radical hysterectomy in cervical cancer: a meta-analysis. J Laparoendosc Adv Surg Tech 25:990–998. 10.1089/lap.2015.0390 10.1089/lap.2015.0390 PMC4691653 26584414

[7] Zhao Y, Hang B, Xiong G-W, Zhang X-W (2017) Laparoscopic radical hysterectomy in early stage cervical cancer: a systematic review and meta-analysis. J Laparoendosc Adv Surg Tech 27:1132–1144. 10.1089/lap.2017.0022 10.1089/lap.2017.0022 28300465

[8] Ramirez PT, Frumovitz M, Pareja R et al (2018) Minimally invasive versus abdominal radical hysterectomy for cervical cancer. N Engl J Med 379(20):1895–1904. 10.1056/NEJMoa1806395 30380365 10.1056/NEJMoa1806395

[9] Nitecki R, Ramirez PT, Frumovitz M et al (2020) Survival after minimally invasive vs open radical hysterectomy for early-stage cervical cancer. JAMA Oncol 6:1019. 10.1001/jamaoncol.2020.1694 32525511 10.1001/jamaoncol.2020.1694 PMC7290695

[10] Hillemanns P, Hertel H, Klapdor R (2020) Radical hysterectomy for early cervical cancer: what shall we do after the LACC trial? Arch Gynecol Obstet 302:289–292. 10.1007/s00404-020-05627-x 32495017 10.1007/s00404-020-05627-x

[11] Kampers J, Gerhardt E, Sibbertsen P, Flock T, Klapdor R, Hertel H, Jentschke M, Hillemanns P (2021) Protective operative techniques in radical hysterectomy in early cervical carcinoma and their influence on disease-free and overall survival: a systematic review and meta-analysis of risk groups. Arch Gynecol Obstet 304(3):577–587. 10.1007/s00404-021-06082-y 34021804 10.1007/s00404-021-06082-y PMC8325671

[12] Limbachiya D, Kumari R (2021) Step-wise technical description of performing ureteric tunnel dissection in laparoscopic radical hysterectomy. Gynecol Minim Invasive Ther 10:215–220 34909378 10.4103/GMIT.GMIT_85_20 PMC8613485

[13] Arbyn M, Weiderpass E, Bruni L, de Sanjosé S, Saraiya M, Ferlay J et al (2020) Estimates of incidence and mortality of cervical cancer in 2018: a worldwide analysis. Lancet Glob Health 8:e191-203 31812369 10.1016/S2214-109X(19)30482-6 PMC7025157

[14] Chua PT, Lee CL, Huang KG (2020) 100% 5-year survival rate in laparoscopic radical hysterectomy for early-stage cervical cancer is an achievable task. Gynecol Minim Invasive Ther 9(2):53 32676279 10.4103/GMIT.GMIT_23_20 PMC7354760

[15] Wenzel HHB, Smolders RGV, Beltman JJ, Lambrechts S, Trum HW, Yigit R et al (2020) Survival of patients with early-stage cervical cancer after abdominal or laparoscopic radical hysterectomy: a nationwide cohort study and literature review. Eur J Cancer 133:14–21 32422504 10.1016/j.ejca.2020.04.006

[16] Togami S, Furuzono N, Mizuno M, Kobayashi H (2025) Comparison of surgical and oncological outcomes between laparoscopic and open surgeries in patients with stage IA1 cervical cancer. Gynecol Minim Invasive Ther 14(2):152–156. 10.4103/gmit.GMIT-D-24-00019 40521580 10.4103/gmit.GMIT-D-24-00019 PMC12165687

[17] Ramirez PT, Robledo KP, Frumovitz M, Pareja R, Ribeiro R, Lopez A et al (2024) LACC trial: final analysis on overall survival comparing open versus minimally invasive radical hysterectomy for early-stage cervical cancer. JCO 42:2741–2746. 10.1200/JCO.23.02335 10.1200/JCO.23.02335 38810208

[18] Melamed A, Margul DJ, Chen L, Keating NL, Del Carmen MG, Yang J et al (2018) Survival after minimally invasive radical hysterectomy for early-stage cervical cancer. N Engl J Med 379(20):1905–1914 30379613 10.1056/NEJMoa1804923 PMC6464372

[19] National Comprehensive Cancer Network. NCCN clinical practice guidelines in oncology: cervical cancer version 1. Accessed April 1, 2020. https://www.nccn.org/professionals/physician_gls/pdf/cervical_blocks.pdf

[20] Nica A, Kim SR, Gien LT, Covens A, Bernardini MQ, Bouchard-Fortier G et al (2020) Survival after minimally invasive surgery in early cervical cancer: is the intra-uterine manipulator to blame? Int J Gynecol Cancer 30(12):1864–1870 33037109 10.1136/ijgc-2020-001816

[21] Printz C (2019) Rethinking a common surgery technique for early cervical cancer: experts are reconsidering the use of minimally invasive radical hysterectomy as a treatment for early cervical cancer after multiple studies found that patients who undergo the procedure by either laparoscopy or robotic surgery have poorer outcomes. Cancer 125(20):3485–3487 31557331 10.1002/cncr.32527

[22] Pyeon SY, Hur YJ, Lee JM (2019) Rethinking the next step after unexpected results associated with minimally invasive radical hysterectomy for early cervical cancer. J Gynecol Oncol 30(2):e43 30740963 10.3802/jgo.2019.30.e43 PMC6393636

[23] Lin F, Pan L, Li L, Li D, Mo L (2014) Effects of a simulated CO2 pneumoperitoneum environment on the proliferation, apoptosis, and metastasis of cervical cancer cells in vitro. Med Sci Monit 20:2497–2503 25436974 10.12659/MSM.891179 PMC4260668

[24] Volz J, Köster S, Spacek Z, Paweletz N (1999) The influence of pneumoperitoneum used in laparoscopic surgery on an intraabdominal tumor growth. Cancer 86:770–774 10463974

[25] Kong TW, Chang SJ, Piao X et al (2016) Patterns of recurrence and survival after abdominal versus laparoscopic/robotic radical hysterectomy in patients with early cervical cancer. J Obstet Gynaecol Res 42:77–86 26554751 10.1111/jog.12840

[26] Gennari P, Tchaikovski S, Mészáros J, Gerken M, Klinkhammer-Schalke M, Toth G et al (2022) Protective effect of pre-operative conization in patients undergoing surgical treatment for early-stage cervical cancer. Gynecol Oncol 166(1):57–60 35618539 10.1016/j.ygyno.2022.05.014

[27] Chacon E, Manzour N, Zanagnolo V, Querleu D, Núñez-Córdoba JM, Martin-Calvo N, SUCCOR Study Group et al (2022) Succor cone study: conization before radical hysterectomy. Int J Gynecol Cancer 32(2):117–124 35039455 10.1136/ijgc-2021-002544

[28] Lago V, Tiermes M, Padilla-Iserte P, Matute L, Gurrea M, Domingo S (2021) Protective maneuver to avoid tumor spillage during laparoscopic radical hysterectomy: vaginal cuff closure. J Minim Invasive Gynecol 28(2):174–175 32540498 10.1016/j.jmig.2020.06.007

[29] Lee CL, Huang KG, Chua PT, Mendoza MCVR, Lee PS, Lai SY (2021) Standardization and experience may influence the survival of laparoscopic radical hysterectomy for cervical cancer. Taiwanese J Obstet Gynecol 60(3):463–467 33966729 10.1016/j.tjog.2021.03.013

